# A comparative analysis of aspatial statistics for detecting racial disparities in cancer mortality rates

**DOI:** 10.1186/1476-072X-6-32

**Published:** 2007-07-24

**Authors:** Pierre Goovaerts, Jaymie R Meliker, Geoffrey M Jacquez

**Affiliations:** 1 BioMedware, Inc, Ann Arbor, MI, USA

## Abstract

**Background:**

Our progress towards the goal of eliminating racial health disparities requires methods for assessing the existence, magnitude, and statistical significance of health disparities. In comparing disease rates, we must account for the unreliability of rates computed for small minority populations and within sparsely populated areas. Furthermore, as the number of geographic units under study increases, we also must account for multiple testing to assure we do not misclassify disparities as present when they actually are not (false positive). To date and to our knowledge, none of the methodologies in current use simultaneously address all of these important needs. And few, if any studies have undertaken a systematic comparison of methods to identify those that are statistically robust and reliable.

**Results:**

We introduced six test statistics for quantifying absolute and relative differences between cancer rates measured in distinct groups (i.e. race or ethnicity). These alternative measures were illustrated using age-adjusted prostate and lung cancer mortality rates for white and black males in 688 counties of the Southeastern US (1970–1994). Statistical performance, including power and proportion of false positives, was investigated in simulation studies that mimic different scenarios for the magnitude and frequency of disparities. Two test statistics, which are based on the difference and ratio of rates, consistently outperformed the other measures. Corrections for multiple testing actually increased misclassification compared with the unadjusted tests and are not recommended. One-tailed tests allowed the researcher to consider a priori hypotheses beyond the basic test that the two rates are different.

**Conclusion:**

The assessment of significant racial disparities across geographic areas is an important tool in guiding cancer control practices, and public health officials must consider the problems of small population size and multiple comparison, and should conduct disparity analyses using both absolute (difference, RD statistic) and relative (ratio, RR statistic) measures. Simple test statistics to assess the significance of rate difference and rate ratio perform well, and their unadjusted *p*-values provide a realistic assessment of the proportion of type I errors (i.e. disparities wrongly declared significant).

## Background

One of the goals of Healthy People 2010 is to "*eliminate health disparities among segments of the population*, *including differences that occur by gender*, *race or ethnicity*, *education or income*, *disability*, *geographic location*, *or sexual orientation*" [1]. This initiative requires the measurement of health disparities, the tracking of differences across various health indicators and geographic areas, and the monitoring of temporal trends. Keppel et al. wrote several methodological reports whose guidelines provide a consistent framework for describing the size and directions of health disparities, facilitating the comparison of disparities over time and across health indicators, geographic areas, and populations [2,3]. They define a disparity as "*The quantity that separates a group from a specified reference point on a particular measure of health that is expressed in terms of a rate*, *percentage*, *mean*, *or some other quantitative measure*". This reference point can be any one of the groups in the population (usually the group with the better health status or the lower risk is chosen), or it could be a standard such as healthy People Target [1].

Cancer data are often aggregated within areas to prevent disclosure of patient identity. These geographic units can span a wide range of scales, such as census units [4], school districts [5], counties [6], and even State Economic Areas [7,8]. Analyses of health disparities in these aggregated datasets can help public health practitioners gain an improved understanding of the causes underlying observed disparities in cancer incidence, mortality, and morbidity, as well as a better assessment of the benefits of current strategies for reducing these disparities.

For comparisons over time or across geographic areas, populations, or indicators, disparities should be measured in both absolute and relative terms since they can lead to contradictory conclusions when not considered together [2]. To illustrate this effect, temporal trends in cervix and prostate cancer mortality were explored using directly age-adjusted cancer mortality rates from the Atlas of Cancer Mortality in the United States [9]. The absolute difference and ratio of rates for black and white populations were calculated for each of the 506 SEA units and five time periods available: 70–74, 75–79, 80–84, 85–89, 90–94. The population-weighted averages of these two statistics are reported in Table 1. The analysis of rate differences suggests a temporal decline in the magnitude of disparities for cervix cancer, while the disparity widens for prostate cancer. This interpretation however ignores the fact that for both races prostate cancer mortality has increased while the mortality has decreased for cervix cancer over the same period. Expressed in terms of relative risks (i.e. ratio), the racial disparities appear to be fairly stable for both cancers across the 25 year time period.

**Table 1:** Population-weighted means of absolute and relative disparity measures computed over 506 State Economic Areas. Calculations are based on age-adjusted mortality rates for black males (BM), white males (WM), black females (BF) and white females (BF).

**Disparity measures**	**1970–1974**	**1975–1979**	**1980–1984**	**1985–1989**	**1990–1994**
**Prostate**					
|BM-WM|	23.49	26.66	27.56	28.47	32.22
BM/WM	2.03	2.14	2.16	2.13	2.22
**Cervix**					
|BF-WF|	8.769	6.816	5.065	5.212	4.074
BF/WF	2.66	2.75	2.50	2.94	2.42

The analysis of health disparities is frequently hampered by the presence of noise in the rates, which is caused by unreliable extreme values estimated from small populations. This effect, known as the "small number problem" [10], is particularly pronounced for minority populations as the analysis proceeds to finer scales, such as neighborhood level commonly used in contextual analysis. Because the observed rates are uncertain, the value of any disparity statistic must also be supplemented by a standard error, allowing the statistical testing of whether the disparity is significant or not [2,3,8]. Standard errors for the disparity statistics are typically computed from the standard errors of rates either through analytical expressions or bootstrap procedures. Keppel et al's report gives an example where these standard errors are calculated using SUDANN [11], a statistical package that adjusts for the effects of the complex design of the National Health Survey. A straightforward alternative, which is implemented in the present paper, is to adopt a Binomial or Poisson distributional model and compute the standard error from the size of the population at risk. For example, the magnitude of differences between sub-population rates can be quantified, and its significance tested, using the large-sample test procedure for equality of two population proportions, also known as t-test [12]. A third option is to capitalize on the spatial correlation between rates measured in neighboring areas to obtain better estimates on the underlying mortality risks and compute the associated standard errors. This spatial analysis can be conducted using a model-based approach, such as Poisson kriging [13,14] or the complex suite of methods developed within the Bayesian framework [15,16].

Another issue related to the analysis of health disparities over small geographic areas is the multiple testing (or multiple comparison) problem caused by the repeated use of statistical tests. As the number of tested areas increases, it becomes increasingly likely that some tests will turn out significant by chance alone (even if the null hypothesis of rate equality is true in all cases). For example, the independent testing of 10 counties under a significance level of 0.05 will lead to a 0.4 probability that at least one test is significant even if none of the 10 counties actually exhibits rate disparity. There are a myriad of approaches to control the rate of these false positives or type I errors. Many of those methods were recently reviewed by Castro and Singer [17] who distinguish two main categories: the experiment-wise or family-wise error rate (FWER) approach (e.g. Bonferroni) and the false discovery rate (FDR) approach. These methods were implemented within the framework of detection of local clusters of high or low values. A small simulation study showed a significant gain in identification of meaningful clusters when using the FDR approach, while FWER tests are too conservative, leading to a large proportion of real clusters being missed. However, the efficiency of these approaches for testing significant disparities across geographic areas has yet to be studied.

Several tests for comparing rates of disease and other health outcomes in specific populations have been developed [2,3], but their application in a spatial context is rare. Critical issues, such as the uncertainty arising from the small size of minority populations or the inflated false discovery rate caused by multiple testing, have not been studied. In general, there is an almost complete lack of comparative evaluations of methods for detecting health disparities, despite the burgeoning analyses in the health science literature [18-21] devoted to the detection of racial disparities. In this study, six test statistics for quantifying absolute and relative differences between disease rates measured in distinct groups (e.g. race or ethnicity) are compared, in combination with four common procedures to correct for multiple testing. Formal statistical power evaluation and proportion of false positives have been carried out using simulation studies that mimic different scenarios for the magnitude and frequency of disparities. In order to generate realistic scenarios, simulations were based on actual geographies, population sizes and cancer mortality rates. Two cancers with a high (i.e. prostate) and low (i.e. lung) level of disparity were chosen and the analysis focused on age-adjusted cancer mortality rates recorded for white and black males in 688 counties of the Southeastern US (1970–1994).

## Methods

### Cancer data sets

Our simulations used directly age-adjusted mortality rates for two cancers with different levels of racial disparity and mortality: lung (low disparity, higher mortality) and prostate (high disparity, lower mortality). Because of the small numbers problem, lower mortality rates are expected to be less reliable estimates of the underlying risk. These data are part of the Atlas of Cancer Mortality in the United States [9]. The analysis was conducted at the county-level, which corresponds to the smallest geography available in the Atlas, and for the only period (1970–1994) where rates for both white (WM) and black males (BM) are reported. The rates were adjusted using the 1970 population pyramid. To reduce the frequency of missing values, the analysis was restricted to 688 counties of the Southeastern US which has the largest minority population.

**Figure 1:**
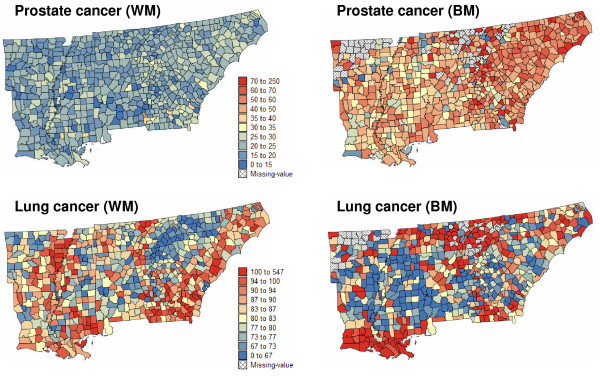
**Maps of age-adjusted prostate and lung cancer mortality rates in 688 counties of the Southeastern US**. The fill color in each county represents the age-adjusted mortality rates per 100,000 person-years recorded over the period 1970–1994. To highlight racial disparities, the same color scale is used for both white males (WM) and black males (BM). Hatched areas correspond to missing data (zero death count).

Figure 1 shows the spatial distribution of mortality rates for both cancers and races. The use of the same color scale for WM and BM rates emphasizes the large magnitude of the disparity for prostate cancer mortality: the population-weighted average is 21.7 per 100,000 person-years for white males and 47.9 per 100,000 person-years for black males. For lung cancer mortality the population-weighted average is 82.7 per 100,000 person-years for white males and 87.3 per 100,000 person-years for black males. For both races, the population at risk was computed as: 100,000 × the total number of deaths from all cancers over the 1970–1994 period divided by the age-adjusted cancer mortality rate; both datasets are available on the NCI website.

### Simulated data sets

An objective assessment of the performance of the different test statistics requires knowledge of the "true" underlying risk maps, which are unknown in practice. Simulation provides a way to generate, for both races, multiple realizations of spatial distributions of cancer mortality rates whose modeled risks are known, and can then be tested for significant disparities using alternative approaches. Results from the different methods can then be interpreted and compared in an experimental setting (the simulations) in which the true magnitude of differences between risk values is known.

For both cancers, the simulation proceeded as follows:

(1) A reference risk map for white males (WM) was generated by a local Bayes smoothing [22] of observed WM mortality rates.

(2) The WM risk map was modified to create a risk map for black males (BM), according to a given scenario for frequency and magnitude of disparities (see below).

(3) For each county and each frequency/magnitude scenario, 50 pairs of WM and BM rates were simulated by random sampling of a Poisson distribution characterized by the county population size and the WM and BM risk values from Steps 1 and 2.

(4) The six disparity statistics were computed for each simulated pair of WM and BM mortality rates, and three different procedures were applied to account for multiple testing (see below).

(5) For each combination of test statistics and multiple testing procedures, performance criteria (e.g. proportions of false negatives and false positives, power of test) were computed by comparing test results with actual risk values.

**Figure 2:**
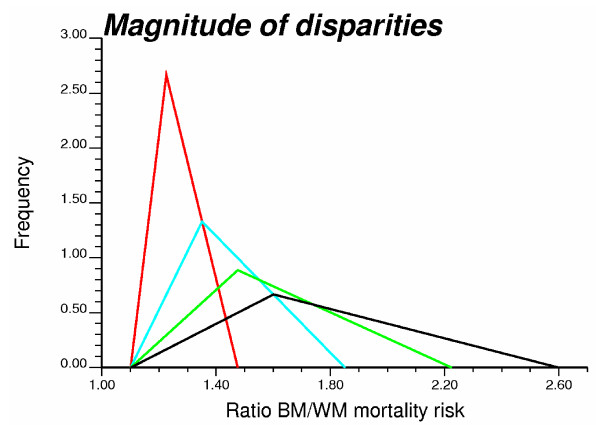
**Four triangular probability distributions used to generate classes of magnitude of disparities in simulation studies**. The values sampled randomly from those distributions are multiplied by the white male (WM) risk values to generate larger risk values for black males (BM) in a random subset of counties.

Four scenarios for the frequency of disparities were considered: 0, 10, 20 and 30% of counties display disparities. These percentages were achieved through a random sampling of the total set of 688 counties. To attenuate the impact of sampling fluctuations, 20 random subsets of the same size were created for each scenario. Once a county has been selected as having disparities, the BM risk value was generated by multiplying the corresponding WM risk by a factor ranging from 1.1 to 2.6. In other words, the mortality risk for black males was modelled as 10 to 160% larger than the risk for white males. The multiplication factor was selected by random sampling of four triangular distributions displayed in Figure 2 and corresponding to four scenarios for the magnitude of disparities. These distributions were constructed using a median value of 1.225, 1.35, 1.475 and 1.60, respectively, and a maximum equal to the median+2(median-minimum).

### Notation

For a given number *N *of geographic units *v*_j _(e.g. counties), denote the observed mortality rates for two non-overlapping categories of individuals (e.g. races, genders) as *z*_1_(*v*_j_) and *z*_2_(*v*_j_). Each rate is computed as the ratio *d*(*v*_j_)/*n*(*v*_j_), where *d*(*v*_j_) is the number of recorded mortality cases and *n*(*v*_j_) is the size of the population at risk. The disease count d(*v*_j_) can be interpreted as a realization of a random variable D(*v*_j_) that follows a Binomial distribution with mean n(*v*_j_)R(*v*_j_) and variance n(*v*_j_)R(*v*_j_)(1 - R(*v*_j_)), where R(*v*_j_) is the mortality risk prevailing over *v*_j_. From the normal approximation to the binomial, one can derive for large samples that the rate z(*v*_j_) is normally distributed asymptotically with mean R(*v*_j_) and variance R(*v*_j_)(1 - R(*v*_j_)).

Without loss of generality, *z*_1 _and *R*_1_will denote the category that is likely to experience the largest population-weighted mean rate (e.g. black males for prostate and lung cancers). The disparity between the two risks *R*_1_(*v*_j_) and *R*_2_(*v*_j_) can be measured either as a difference [*R*_1_(*v*_j_)-*R*_2_(*v*_j_)] or a ratio *R*_1_(*v*_j_)/*R*_2_(*v*_j_). Following Lachin [23], these two quantities will be referred to as risk difference (RD) and relative risk (RR).

### Two-tailed tests for risk difference

The null and alternative hypotheses for testing the equality of risks for two ethnic groups are:

H_0 _: *R*_1_(*v*_*j*_) = *R*_2_(*v*_*j*_)

H_alt _: *R*_1_(*v*_*j*_) ≠ *R*_2_(*v*_*j*_)

which can be rewritten in terms of risk differences as:

H_0 _: |*RD*(*v*_*j*_)| = 0

H_alt _: |*RD*(*v*_*j*_)| ≠ 0

This test is two-tailed in that a difference is declared significant either if *R*_1_(*v*_j_) is sufficiently greater than *R*_2_(*v*_j_) or if *R*_1_(*v*_j_) is sufficiently less than *R*_2_(*v*_j_). In other words, the sign of the difference does not matter and the test is conducted on the absolute value of the RD statistic. Following Fleiss [24] and Lachim [23] the following four test statistics, which all follow a standard normal distribution N(0, 1), are available:

DispI(vj)=|z1(vj)−z2(vj)|z¯j(1−z¯j)[1n1(vj)+1n2(vj)]
 MathType@MTEF@5@5@+=feaafiart1ev1aaatCvAUfKttLearuWrP9MDH5MBPbIqV92AaeXatLxBI9gBaebbnrfifHhDYfgasaacH8akY=wiFfYdH8Gipec8Eeeu0xXdbba9frFj0=OqFfea0dXdd9vqai=hGuQ8kuc9pgc9s8qqaq=dirpe0xb9q8qiLsFr0=vr0=vr0dc8meaabaqaciaacaGaaeqabaqabeGadaaakeaacqqGebarcqqGPbqAcqqGZbWCcqqGWbaCdaWgaaWcbaGaeeysaKeabeaakiabcIcaOiabdAha2naaBaaaleaacqWGQbGAaeqaaOGaeiykaKIaeyypa0ZaaSaaaeaadaabdaqaaiabdQha6naaBaaaleaacqaIXaqmaeqaaOGaeiikaGIaemODay3aaSbaaSqaaiabdQgaQbqabaGccqGGPaqkcqGHsislcqWG6bGEdaWgaaWcbaGaeGOmaidabeaakiabcIcaOiabdAha2naaBaaaleaacqWGQbGAaeqaaOGaeiykaKcacaGLhWUaayjcSdaabaWaaOaaaeaacuWG6bGEgaqeamaaBaaaleaacqWGQbGAaeqaaOGaeiikaGIaeGymaeJaeyOeI0IafmOEaONbaebadaWgaaWcbaGaemOAaOgabeaakiabcMcaPmaadmaabaWaaSaaaeaacqaIXaqmaeaacqWGUbGBdaWgaaWcbaGaeGymaedabeaakiabcIcaOiabdAha2naaBaaaleaacqWGQbGAaeqaaOGaeiykaKcaaiabgUcaRmaalaaabaGaeGymaedabaGaemOBa42aaSbaaSqaaiabikdaYaqabaGccqGGOaakcqWG2bGDdaWgaaWcbaGaemOAaOgabeaakiabcMcaPaaaaiaawUfacaGLDbaaaSqabaaaaaaa@692C@

where z¯
 MathType@MTEF@5@5@+=feaafiart1ev1aaatCvAUfKttLearuWrP9MDH5MBPbIqV92AaeXatLxBI9gBaebbnrfifHhDYfgasaacH8akY=wiFfYdH8Gipec8Eeeu0xXdbba9frFj0=OqFfea0dXdd9vqai=hGuQ8kuc9pgc9s8qqaq=dirpe0xb9q8qiLsFr0=vr0=vr0dc8meaabaqaciaacaGaaeqabaqabeGadaaakeaacuWG6bGEgaqeaaaa@2E41@_*j *_is the population-weighted average of rates:

z¯j=n1(vj)z1(vj)+n2(vj)z2(vj)n1(vj)+n2(vj)
 MathType@MTEF@5@5@+=feaafiart1ev1aaatCvAUfKttLearuWrP9MDH5MBPbIqV92AaeXatLxBI9gBaebbnrfifHhDYfgasaacH8akY=wiFfYdH8Gipec8Eeeu0xXdbba9frFj0=OqFfea0dXdd9vqai=hGuQ8kuc9pgc9s8qqaq=dirpe0xb9q8qiLsFr0=vr0=vr0dc8meaabaqaciaacaGaaeqabaqabeGadaaakeaacuWG6bGEgaqeamaaBaaaleaacqWGQbGAaeqaaOGaeyypa0ZaaSaaaeaacqWGUbGBdaWgaaWcbaGaeGymaedabeaakiabcIcaOiabdAha2naaBaaaleaacqWGQbGAaeqaaOGaeiykaKIaemOEaO3aaSbaaSqaaiabigdaXaqabaGccqGGOaakcqWG2bGDdaWgaaWcbaGaemOAaOgabeaakiabcMcaPiabgUcaRiabd6gaUnaaBaaaleaacqaIYaGmaeqaaOGaeiikaGIaemODay3aaSbaaSqaaiabdQgaQbqabaGccqGGPaqkcqWG6bGEdaWgaaWcbaGaeGOmaidabeaakiabcIcaOiabdAha2naaBaaaleaacqWGQbGAaeqaaOGaeiykaKcabaGaemOBa42aaSbaaSqaaiabigdaXaqabaGccqGGOaakcqWG2bGDdaWgaaWcbaGaemOAaOgabeaakiabcMcaPiabgUcaRiabd6gaUnaaBaaaleaacqaIYaGmaeqaaOGaeiikaGIaemODay3aaSbaaSqaaiabdQgaQbqabaGccqGGPaqkaaaaaa@5E82@

The second statistic is more general in that there is no restriction on the values of the two rates, i.e. they are not averaged in order to compute the denominator:

DispII(vj)=|z1(vj)−z2(vj)|z1(vj)(1−z1(vj))n1(vj)+z2(vj)(1−z2(vj))n2(vj)
 MathType@MTEF@5@5@+=feaafiart1ev1aaatCvAUfKttLearuWrP9MDH5MBPbIqV92AaeXatLxBI9gBaebbnrfifHhDYfgasaacH8akY=wiFfYdH8Gipec8Eeeu0xXdbba9frFj0=OqFfea0dXdd9vqai=hGuQ8kuc9pgc9s8qqaq=dirpe0xb9q8qiLsFr0=vr0=vr0dc8meaabaqaciaacaGaaeqabaqabeGadaaakeaacqqGebarcqqGPbqAcqqGZbWCcqqGWbaCdaWgaaWcbaGaeeysaKKaeeysaKeabeaakiabcIcaOiabdAha2naaBaaaleaacqWGQbGAaeqaaOGaeiykaKIaeyypa0ZaaSaaaeaadaabdaqaaiabdQha6naaBaaaleaacqaIXaqmaeqaaOGaeiikaGIaemODay3aaSbaaSqaaiabdQgaQbqabaGccqGGPaqkcqGHsislcqWG6bGEdaWgaaWcbaGaeGOmaidabeaakiabcIcaOiabdAha2naaBaaaleaacqWGQbGAaeqaaOGaeiykaKcacaGLhWUaayjcSdaabaWaaOaaaeaadaWcaaqaaiabdQha6naaBaaaleaacqaIXaqmaeqaaOGaeiikaGIaemODay3aaSbaaSqaaiabdQgaQbqabaGccqGGPaqkcqGGOaakcqaIXaqmcqGHsislcqWG6bGEdaWgaaWcbaGaeGymaedabeaakiabcIcaOiabdAha2naaBaaaleaacqWGQbGAaeqaaOGaeiykaKIaeiykaKcabaGaemOBa42aaSbaaSqaaiabigdaXaqabaGccqGGOaakcqWG2bGDdaWgaaWcbaGaemOAaOgabeaakiabcMcaPaaacqGHRaWkdaWcaaqaaiabdQha6naaBaaaleaacqaIYaGmaeqaaOGaeiikaGIaemODay3aaSbaaSqaaiabdQgaQbqabaGccqGGPaqkcqGGOaakcqaIXaqmcqGHsislcqWG6bGEdaWgaaWcbaGaeGOmaidabeaakiabcIcaOiabdAha2naaBaaaleaacqWGQbGAaeqaaOGaeiykaKIaeiykaKcabaGaemOBa42aaSbaaSqaaiabikdaYaqabaGccqGGOaakcqWG2bGDdaWgaaWcbaGaemOAaOgabeaakiabcMcaPaaaaSqabaaaaaaa@812A@

The last two statistics are obtained by subtracting from the numerator the following Yates' correction for continuity:

c(vj)=0.5[1n1(vj)+1n2(vj)]
 MathType@MTEF@5@5@+=feaafiart1ev1aaatCvAUfKttLearuWrP9MDH5MBPbIqV92AaeXatLxBI9gBaebbnrfifHhDYfgasaacH8akY=wiFfYdH8Gipec8Eeeu0xXdbba9frFj0=OqFfea0dXdd9vqai=hGuQ8kuc9pgc9s8qqaq=dirpe0xb9q8qiLsFr0=vr0=vr0dc8meaabaqaciaacaGaaeqabaqabeGadaaakeaacqWGJbWycqGGOaakcqWG2bGDdaWgaaWcbaGaemOAaOgabeaakiabcMcaPiabg2da9iabicdaWiabc6caUiabiwda1maadmaabaWaaSaaaeaacqaIXaqmaeaacqWGUbGBdaWgaaWcbaGaeGymaedabeaakiabcIcaOiabdAha2naaBaaaleaacqWGQbGAaeqaaOGaeiykaKcaaiabgUcaRmaalaaabaGaeGymaedabaGaemOBa42aaSbaaSqaaiabikdaYaqabaGccqGGOaakcqWG2bGDdaWgaaWcbaGaemOAaOgabeaakiabcMcaPaaaaiaawUfacaGLDbaaaaa@49E5@

This correction accounts for the fact that a continuous distribution (i.e. normal) is used to represent the discrete distribution of sample frequencies. The corrected statistics are expressed as follows:

DispIII(vj)=|z1(vj)−z2(vj)|−c(vj)z¯j(1−z¯j)[1n1(vj)+1n2(vj)]
 MathType@MTEF@5@5@+=feaafiart1ev1aaatCvAUfKttLearuWrP9MDH5MBPbIqV92AaeXatLxBI9gBaebbnrfifHhDYfgasaacH8akY=wiFfYdH8Gipec8Eeeu0xXdbba9frFj0=OqFfea0dXdd9vqai=hGuQ8kuc9pgc9s8qqaq=dirpe0xb9q8qiLsFr0=vr0=vr0dc8meaabaqaciaacaGaaeqabaqabeGadaaakeaacqqGebarcqqGPbqAcqqGZbWCcqqGWbaCdaWgaaWcbaGaeeysaKKaeeysaKKaeeysaKeabeaakiabcIcaOiabdAha2naaBaaaleaacqWGQbGAaeqaaOGaeiykaKIaeyypa0ZaaSaaaeaadaabdaqaaiabdQha6naaBaaaleaacqaIXaqmaeqaaOGaeiikaGIaemODay3aaSbaaSqaaiabdQgaQbqabaGccqGGPaqkcqGHsislcqWG6bGEdaWgaaWcbaGaeGOmaidabeaakiabcIcaOiabdAha2naaBaaaleaacqWGQbGAaeqaaOGaeiykaKcacaGLhWUaayjcSdGaeyOeI0Iaem4yamMaeiikaGIaemODay3aaSbaaSqaaiabdQgaQbqabaGccqGGPaqkaeaadaGcaaqaaiqbdQha6zaaraWaaSbaaSqaaiabdQgaQbqabaGccqGGOaakcqaIXaqmcqGHsislcuWG6bGEgaqeamaaBaaaleaacqWGQbGAaeqaaOGaeiykaKYaamWaaeaadaWcaaqaaiabigdaXaqaaiabd6gaUnaaBaaaleaacqaIXaqmaeqaaOGaeiikaGIaemODay3aaSbaaSqaaiabdQgaQbqabaGccqGGPaqkaaGaey4kaSYaaSaaaeaacqaIXaqmaeaacqWGUbGBdaWgaaWcbaGaeGOmaidabeaakiabcIcaOiabdAha2naaBaaaleaacqWGQbGAaeqaaOGaeiykaKcaaaGaay5waiaaw2faaaWcbeaaaaaaaa@7254@

DispIV(vj)=|z1(vj)−z2(vj)|−c(vj)z1(vj)(1−z1(vj))n1(vj)+z2(vj)(1−z2(vj))n2(vj)
 MathType@MTEF@5@5@+=feaafiart1ev1aaatCvAUfKttLearuWrP9MDH5MBPbIqV92AaeXatLxBI9gBaebbnrfifHhDYfgasaacH8akY=wiFfYdH8Gipec8Eeeu0xXdbba9frFj0=OqFfea0dXdd9vqai=hGuQ8kuc9pgc9s8qqaq=dirpe0xb9q8qiLsFr0=vr0=vr0dc8meaabaqaciaacaGaaeqabaqabeGadaaakeaacqqGebarcqqGPbqAcqqGZbWCcqqGWbaCdaWgaaWcbaGaeeysaKKaeeOvayfabeaakiabcIcaOiabdAha2naaBaaaleaacqWGQbGAaeqaaOGaeiykaKIaeyypa0ZaaSaaaeaadaabdaqaaiabdQha6naaBaaaleaacqaIXaqmaeqaaOGaeiikaGIaemODay3aaSbaaSqaaiabdQgaQbqabaGccqGGPaqkcqGHsislcqWG6bGEdaWgaaWcbaGaeGOmaidabeaakiabcIcaOiabdAha2naaBaaaleaacqWGQbGAaeqaaOGaeiykaKcacaGLhWUaayjcSdGaeyOeI0Iaem4yamMaeiikaGIaemODay3aaSbaaSqaaiabdQgaQbqabaGccqGGPaqkaeaadaGcaaqaamaalaaabaGaemOEaO3aaSbaaSqaaiabigdaXaqabaGccqGGOaakcqWG2bGDdaWgaaWcbaGaemOAaOgabeaakiabcMcaPiabcIcaOiabigdaXiabgkHiTiabdQha6naaBaaaleaacqaIXaqmaeqaaOGaeiikaGIaemODay3aaSbaaSqaaiabdQgaQbqabaGccqGGPaqkcqGGPaqkaeaacqWGUbGBdaWgaaWcbaGaeGymaedabeaakiabcIcaOiabdAha2naaBaaaleaacqWGQbGAaeqaaOGaeiykaKcaaiabgUcaRmaalaaabaGaemOEaO3aaSbaaSqaaiabikdaYaqabaGccqGGOaakcqWG2bGDdaWgaaWcbaGaemOAaOgabeaakiabcMcaPiabcIcaOiabigdaXiabgkHiTiabdQha6naaBaaaleaacqaIYaGmaeqaaOGaeiikaGIaemODay3aaSbaaSqaaiabdQgaQbqabaGccqGGPaqkcqGGPaqkaeaacqWGUbGBdaWgaaWcbaGaeGOmaidabeaakiabcIcaOiabdAha2naaBaaaleaacqWGQbGAaeqaaOGaeiykaKcaaaWcbeaaaaaaaa@883A@

### Two-tailed tests for risk ratio

The null and alternative hypotheses for testing the relative equality of risks for two ethnic groups are expressed in terms of ratios as:

H_0 _: ⌊*R*_1_(*v*_*j*_)/*R*_2_(*v*_*j*_)⌋ = *RR*(*v*_*j*_) = 1

H_alt _: [*R*_1_(*v*_*j*_)/*R*_2_(*v*_*j*_)] = *RR*(*v*_*j*_) ≠ 1

For the risk ratios, the domain is not symmetric about the null value. Thus, the large sample distributions are better approximated using the log transformation. As for the risk difference, depending on the restrictions imposed on the values of the two rates, two types of statistics can be defined:

DispV(vj)=log⁡(z1(vj)z2(vj))(1−z¯j)z¯j[1n1(vj)+1n2(vj)]
 MathType@MTEF@5@5@+=feaafiart1ev1aaatCvAUfKttLearuWrP9MDH5MBPbIqV92AaeXatLxBI9gBaebbnrfifHhDYfgasaacH8akY=wiFfYdH8Gipec8Eeeu0xXdbba9frFj0=OqFfea0dXdd9vqai=hGuQ8kuc9pgc9s8qqaq=dirpe0xb9q8qiLsFr0=vr0=vr0dc8meaabaqaciaacaGaaeqabaqabeGadaaakeaacqqGebarcqqGPbqAcqqGZbWCcqqGWbaCdaWgaaWcbaGaeeOvayfabeaakiabcIcaOiabdAha2naaBaaaleaacqWGQbGAaeqaaOGaeiykaKIaeyypa0ZaaSaaaeaacyGGSbaBcqGGVbWBcqGGNbWzdaqadaqaamaalaaabaGaemOEaO3aaSbaaSqaaiabigdaXaqabaGccqGGOaakcqWG2bGDdaWgaaWcbaGaemOAaOgabeaakiabcMcaPaqaaiabdQha6naaBaaaleaacqaIYaGmaeqaaOGaeiikaGIaemODay3aaSbaaSqaaiabdQgaQbqabaGccqGGPaqkaaaacaGLOaGaayzkaaaabaWaaOaaaeaadaWcaaqaaiabcIcaOiabigdaXiabgkHiTiqbdQha6zaaraWaaSbaaSqaaiabdQgaQbqabaGccqGGPaqkaeaacuWG6bGEgaqeamaaBaaaleaacqWGQbGAaeqaaaaakmaadmaabaWaaSaaaeaacqaIXaqmaeaacqWGUbGBdaWgaaWcbaGaeGymaedabeaakiabcIcaOiabdAha2naaBaaaleaacqWGQbGAaeqaaOGaeiykaKcaaiabgUcaRmaalaaabaGaeGymaedabaGaemOBa42aaSbaaSqaaiabikdaYaqabaGccqGGOaakcqWG2bGDdaWgaaWcbaGaemOAaOgabeaakiabcMcaPaaaaiaawUfacaGLDbaaaSqabaaaaaaa@6AFE@

DispVI(vj)=log⁡(z1(vj)z2(vj))(1−z1(vj))n1(vj)z1(vj)+(1−z2(vj))n2(vj)z2(vj)
 MathType@MTEF@5@5@+=feaafiart1ev1aaatCvAUfKttLearuWrP9MDH5MBPbIqV92AaeXatLxBI9gBaebbnrfifHhDYfgasaacH8akY=wiFfYdH8Gipec8Eeeu0xXdbba9frFj0=OqFfea0dXdd9vqai=hGuQ8kuc9pgc9s8qqaq=dirpe0xb9q8qiLsFr0=vr0=vr0dc8meaabaqaciaacaGaaeqabaqabeGadaaakeaacqqGebarcqqGPbqAcqqGZbWCcqqGWbaCdaWgaaWcbaGaeeOvayLaeeysaKeabeaakiabcIcaOiabdAha2naaBaaaleaacqWGQbGAaeqaaOGaeiykaKIaeyypa0ZaaSaaaeaacyGGSbaBcqGGVbWBcqGGNbWzdaqadaqaamaalaaabaGaemOEaO3aaSbaaSqaaiabigdaXaqabaGccqGGOaakcqWG2bGDdaWgaaWcbaGaemOAaOgabeaakiabcMcaPaqaaiabdQha6naaBaaaleaacqaIYaGmaeqaaOGaeiikaGIaemODay3aaSbaaSqaaiabdQgaQbqabaGccqGGPaqkaaaacaGLOaGaayzkaaaabaWaaOaaaeaadaWcaaqaaiabcIcaOiabigdaXiabgkHiTiabdQha6naaBaaaleaacqaIXaqmaeqaaOGaeiikaGIaemODay3aaSbaaSqaaiabdQgaQbqabaGccqGGPaqkcqGGPaqkaeaacqWGUbGBdaWgaaWcbaGaeGymaedabeaakiabcIcaOiabdAha2naaBaaaleaacqWGQbGAaeqaaOGaeiykaKIaemOEaO3aaSbaaSqaaiabigdaXaqabaGccqGGOaakcqWG2bGDdaWgaaWcbaGaemOAaOgabeaakiabcMcaPaaacqGHRaWkdaWcaaqaaiabcIcaOiabigdaXiabgkHiTiabdQha6naaBaaaleaacqaIYaGmaeqaaOGaeiikaGIaemODay3aaSbaaSqaaiabdQgaQbqabaGccqGGPaqkcqGGPaqkaeaacqWGUbGBdaWgaaWcbaGaeGOmaidabeaakiabcIcaOiabdAha2naaBaaaleaacqWGQbGAaeqaaOGaeiykaKIaemOEaO3aaSbaaSqaaiabikdaYaqabaGccqGGOaakcqWG2bGDdaWgaaWcbaGaemOAaOgabeaakiabcMcaPaaaaSqabaaaaaaa@82EC@

where z¯
 MathType@MTEF@5@5@+=feaafiart1ev1aaatCvAUfKttLearuWrP9MDH5MBPbIqV92AaeXatLxBI9gBaebbnrfifHhDYfgasaacH8akY=wiFfYdH8Gipec8Eeeu0xXdbba9frFj0=OqFfea0dXdd9vqai=hGuQ8kuc9pgc9s8qqaq=dirpe0xb9q8qiLsFr0=vr0=vr0dc8meaabaqaciaacaGaaeqabaqabeGadaaakeaacuWG6bGEgaqeaaaa@2E41@_*j *_is the population-weighted average of rates defined earlier. Both test statistics follow a standard normal distribution.

### One-tailed tests

In some situations such as prostate cancer mortality, the disparity between rates is so large and systematic that two-tailed tests are not highly informative: they simply confirm that the rates for black and white males are significantly different over most of the counties. A more interesting hypothesis, such as the exceedence of a particular disparity threshold Δ_0_, can be tested using the following one-tailed test:

H_0 _: *RD*(*v*_*j*_) = Δ_0_

H_alt _: *RD*(*v*_*j*_) > Δ_0_

For example, setting Δ_0 _= 10 would lead to the identification of counties where the BM mortality rate exceeds WM rate by an amount that is significantly greater than 10 deaths/100,000 habitants. The statistics introduced for the two-tailed tests can be easily adapted to the new type of assumption; e.g. for the first two statistics:

DispI(vj)=(z1(vj)−z2(vj))−Δ0(z¯j+Δ0)(1−(z¯j+Δ0))n1(vj)+z¯j(1−z¯j)n2(vj)
 MathType@MTEF@5@5@+=feaafiart1ev1aaatCvAUfKttLearuWrP9MDH5MBPbIqV92AaeXatLxBI9gBaebbnrfifHhDYfgasaacH8akY=wiFfYdH8Gipec8Eeeu0xXdbba9frFj0=OqFfea0dXdd9vqai=hGuQ8kuc9pgc9s8qqaq=dirpe0xb9q8qiLsFr0=vr0=vr0dc8meaabaqaciaacaGaaeqabaqabeGadaaakeaacqqGebarcqqGPbqAcqqGZbWCcqqGWbaCdaWgaaWcbaGaeeysaKeabeaakiabcIcaOiabdAha2naaBaaaleaacqWGQbGAaeqaaOGaeiykaKIaeyypa0ZaaSaaaeaadaqadaqaaiabdQha6naaBaaaleaacqaIXaqmaeqaaOGaeiikaGIaemODay3aaSbaaSqaaiabdQgaQbqabaGccqGGPaqkcqGHsislcqWG6bGEdaWgaaWcbaGaeGOmaidabeaakiabcIcaOiabdAha2naaBaaaleaacqWGQbGAaeqaaOGaeiykaKcacaGLOaGaayzkaaGaeyOeI0IaeuiLdq0aaSbaaSqaaiabicdaWaqabaaakeaadaGcaaqaamaalaaabaGaeiikaGIafmOEaONbaebadaWgaaWcbaGaemOAaOgabeaakiabgUcaRiabfs5aenaaBaaaleaacqaIWaamaeqaaOGaeiykaKIaeiikaGIaeGymaeJaeyOeI0IaeiikaGIafmOEaONbaebadaWgaaWcbaGaemOAaOgabeaakiabgUcaRiabfs5aenaaBaaaleaacqaIWaamaeqaaOGaeiykaKIaeiykaKcabaGaemOBa42aaSbaaSqaaiabigdaXaqabaGccqGGOaakcqWG2bGDdaWgaaWcbaGaemOAaOgabeaakiabcMcaPaaacqGHRaWkdaWcaaqaaiqbdQha6zaaraWaaSbaaSqaaiabdQgaQbqabaGccqGGOaakcqaIXaqmcqGHsislcuWG6bGEgaqeamaaBaaaleaacqWGQbGAaeqaaOGaeiykaKcabaGaemOBa42aaSbaaSqaaiabikdaYaqabaGccqGGOaakcqWG2bGDdaWgaaWcbaGaemOAaOgabeaakiabcMcaPaaaaSqabaaaaaaa@7B53@

DispII(vj)=(z1(vj)−z2(vj))−Δ0zj(vj)(1−z1(vj))n1(vj)+z2(vj)(1−z2(vj))n2(vj)
 MathType@MTEF@5@5@+=feaafiart1ev1aaatCvAUfKttLearuWrP9MDH5MBPbIqV92AaeXatLxBI9gBaebbnrfifHhDYfgasaacH8akY=wiFfYdH8Gipec8Eeeu0xXdbba9frFj0=OqFfea0dXdd9vqai=hGuQ8kuc9pgc9s8qqaq=dirpe0xb9q8qiLsFr0=vr0=vr0dc8meaabaqaciaacaGaaeqabaqabeGadaaakeaacqqGebarcqqGPbqAcqqGZbWCcqqGWbaCdaWgaaWcbaGaeeysaKKaeeysaKeabeaakiabcIcaOiabdAha2naaBaaaleaacqWGQbGAaeqaaOGaeiykaKIaeyypa0ZaaSaaaeaadaqadaqaaiabdQha6naaBaaaleaacqaIXaqmaeqaaOGaeiikaGIaemODay3aaSbaaSqaaiabdQgaQbqabaGccqGGPaqkcqGHsislcqWG6bGEdaWgaaWcbaGaeGOmaidabeaakiabcIcaOiabdAha2naaBaaaleaacqWGQbGAaeqaaOGaeiykaKcacaGLOaGaayzkaaGaeyOeI0IaeuiLdq0aaSbaaSqaaiabicdaWaqabaaakeaadaGcaaqaamaalaaabaGaemOEaO3aaSbaaSqaaiabdQgaQbqabaGccqGGOaakcqWG2bGDdaWgaaWcbaGaemOAaOgabeaakiabcMcaPiabcIcaOiabigdaXiabgkHiTiabdQha6naaBaaaleaacqaIXaqmaeqaaOGaeiikaGIaemODay3aaSbaaSqaaiabdQgaQbqabaGccqGGPaqkcqGGPaqkaeaacqWGUbGBdaWgaaWcbaGaeGymaedabeaakiabcIcaOiabdAha2naaBaaaleaacqWGQbGAaeqaaOGaeiykaKcaaiabgUcaRmaalaaabaGaemOEaO3aaSbaaSqaaiabikdaYaqabaGccqGGOaakcqWG2bGDdaWgaaWcbaGaemOAaOgabeaakiabcMcaPiabcIcaOiabigdaXiabgkHiTiabdQha6naaBaaaleaacqaIYaGmaeqaaOGaeiikaGIaemODay3aaSbaaSqaaiabdQgaQbqabaGccqGGPaqkcqGGPaqkaeaacqWGUbGBdaWgaaWcbaGaeGOmaidabeaakiabcIcaOiabdAha2naaBaaaleaacqWGQbGAaeqaaOGaeiykaKcaaaWcbeaaaaaaaa@8375@

Note that even when Δ_0 _= 0 one-tailed and two-tailed tests might not lead to the same conclusion for the same significance level α. One-tailed tests are more powerful than two-tailed tests since it is easier to reject the null hypothesis of equality of rates when the rates differ in the direction specified by the alternative hypothesis. The trade-off cost is that the investigator needs to formulate a priori assumptions regarding the direction (i.e. sign) of the difference between rates. However, the scientific importance of detecting a difference in the unexpected direction (e.g. prostate cancer mortality larger for white males than black males) may exceed yet another confirmation of the difference being in the expected direction [24].

Using a similar reasoning, one-tailed tests can be formulated for the ratio of risks:

H_0 _: *RR*(*v*_*j*_) = (1 + Δ_0_)

H_alt _: *RR*(*v*_*j*_) > (1 + Δ_0_)

where the disparity threshold Δ_0 _is now expressed as a proportion; e.g. 0.2 to test whether the BM mortality rate is significantly greater than 120% of the WM rate. The statistics introduced for the two-tailed tests can be easily adapted to the new type of assumption; e.g.:

DispV(vj)=log⁡(z1(vj)z2(vj))−log⁡(1+Δ0)(1−z¯j×(1+Δ0))n1(vj)z¯j×(1+Δ0)+(1−z¯j)n2(vj)z¯j
 MathType@MTEF@5@5@+=feaafiart1ev1aaatCvAUfKttLearuWrP9MDH5MBPbIqV92AaeXatLxBI9gBaebbnrfifHhDYfgasaacH8akY=wiFfYdH8Gipec8Eeeu0xXdbba9frFj0=OqFfea0dXdd9vqai=hGuQ8kuc9pgc9s8qqaq=dirpe0xb9q8qiLsFr0=vr0=vr0dc8meaabaqaciaacaGaaeqabaqabeGadaaakeaacqqGebarcqqGPbqAcqqGZbWCcqqGWbaCdaWgaaWcbaGaeeOvayfabeaakiabcIcaOiabdAha2naaBaaaleaacqWGQbGAaeqaaOGaeiykaKIaeyypa0ZaaSaaaeaacyGGSbaBcqGGVbWBcqGGNbWzdaqadaqaamaalaaabaGaemOEaO3aaSbaaSqaaiabigdaXaqabaGccqGGOaakcqWG2bGDdaWgaaWcbaGaemOAaOgabeaakiabcMcaPaqaaiabdQha6naaBaaaleaacqaIYaGmaeqaaOGaeiikaGIaemODay3aaSbaaSqaaiabdQgaQbqabaGccqGGPaqkaaaacaGLOaGaayzkaaGaeyOeI0IagiiBaWMaei4Ba8Maei4zaCMaeiikaGIaeGymaeJaey4kaSIaeuiLdq0aaSbaaSqaaiabicdaWaqabaGccqGGPaqkaeaadaGcaaqaamaalaaabaGaeiikaGIaeGymaeJaeyOeI0IafmOEaONbaebadaWgaaWcbaGaemOAaOgabeaakiabgEna0oaabmaabaGaeGymaeJaey4kaSIaeuiLdq0aaSbaaSqaaiabicdaWaqabaaakiaawIcacaGLPaaacqGGPaqkaeaacqWGUbGBdaWgaaWcbaGaeGymaedabeaakiabcIcaOiabdAha2naaBaaaleaacqWGQbGAaeqaaOGaeiykaKIafmOEaONbaebadaWgaaWcbaGaemOAaOgabeaakiabgEna0oaabmaabaGaeGymaeJaey4kaSIaeuiLdq0aaSbaaSqaaiabicdaWaqabaaakiaawIcacaGLPaaaaaGaey4kaSYaaSaaaeaacqGGOaakcqaIXaqmcqGHsislcuWG6bGEgaqeamaaBaaaleaacqWGQbGAaeqaaOGaeiykaKcabaGaemOBa42aaSbaaSqaaiabikdaYaqabaGccqGGOaakcqWG2bGDdaWgaaWcbaGaemOAaOgabeaakiabcMcaPiqbdQha6zaaraWaaSbaaSqaaiabdQgaQbqabaaaaaqabaaaaaaa@8BF7@

DispVI(vj)=log⁡(z1(vj)z2(vj))−log⁡(1+Δ0)(1−z1(vj))n1(vj)z1(vj)+(1−z2(vj))n2(vj)z2(vj)
 MathType@MTEF@5@5@+=feaafiart1ev1aaatCvAUfKttLearuWrP9MDH5MBPbIqV92AaeXatLxBI9gBaebbnrfifHhDYfgasaacH8akY=wiFfYdH8Gipec8Eeeu0xXdbba9frFj0=OqFfea0dXdd9vqai=hGuQ8kuc9pgc9s8qqaq=dirpe0xb9q8qiLsFr0=vr0=vr0dc8meaabaqaciaacaGaaeqabaqabeGadaaakeaacqqGebarcqqGPbqAcqqGZbWCcqqGWbaCdaWgaaWcbaGaeeOvayLaeeysaKeabeaakiabcIcaOiabdAha2naaBaaaleaacqWGQbGAaeqaaOGaeiykaKIaeyypa0ZaaSaaaeaacyGGSbaBcqGGVbWBcqGGNbWzdaqadaqaamaalaaabaGaemOEaO3aaSbaaSqaaiabigdaXaqabaGccqGGOaakcqWG2bGDdaWgaaWcbaGaemOAaOgabeaakiabcMcaPaqaaiabdQha6naaBaaaleaacqaIYaGmaeqaaOGaeiikaGIaemODay3aaSbaaSqaaiabdQgaQbqabaGccqGGPaqkaaaacaGLOaGaayzkaaGaeyOeI0IagiiBaWMaei4Ba8Maei4zaCMaeiikaGIaeGymaeJaey4kaSIaeuiLdq0aaSbaaSqaaiabicdaWaqabaGccqGGPaqkaeaadaGcaaqaamaalaaabaGaeiikaGIaeGymaeJaeyOeI0IaemOEaO3aaSbaaSqaaiabigdaXaqabaGccqGGOaakcqWG2bGDdaWgaaWcbaGaemOAaOgabeaakiabcMcaPiabcMcaPaqaaiabd6gaUnaaBaaaleaacqaIXaqmaeqaaOGaeiikaGIaemODay3aaSbaaSqaaiabdQgaQbqabaGccqGGPaqkcqWG6bGEdaWgaaWcbaGaeGymaedabeaakiabcIcaOiabdAha2naaBaaaleaacqWGQbGAaeqaaOGaeiykaKcaaiabgUcaRmaalaaabaGaeiikaGIaeGymaeJaeyOeI0IaemOEaO3aaSbaaSqaaiabikdaYaqabaGccqGGOaakcqWG2bGDdaWgaaWcbaGaemOAaOgabeaakiabcMcaPiabcMcaPaqaaiabd6gaUnaaBaaaleaacqaIYaGmaeqaaOGaeiikaGIaemODay3aaSbaaSqaaiabdQgaQbqabaGccqGGPaqkcqWG6bGEdaWgaaWcbaGaeGOmaidabeaakiabcIcaOiabdAha2naaBaaaleaacqWGQbGAaeqaaOGaeiykaKcaaaWcbeaaaaaaaa@8E05@

Once again, if Δ_0 _= 0 the one-tailed and two-tailed test statistics are the same, but since the significance level α is assigned to a single direction in the one-tailed test it will be more powerful than the corresponding two-tailed test.

### Multiple testing corrections

Regardless of the type of hypothesis under consideration, once the test statistic Disp(v_j_) has been computed, its significance must be evaluated. This step typically requires computing the probability of obtaining a result as extreme as the test statistic by chance alone, under the null hypothesis of equality of rates. This probability, called the *p*-value of the test, is obtained by comparing the test statistic to its expected distribution under the null hypothesis, which is a standard normal distribution for all of the tests considered. One thus rejects the null hypothesis if the *p*-value exceeds the significance level α which is typically set to 0.05 (significant difference) or 0.01 (highly significant difference). In other words, one rejects the null hypothesis if it appears very unlikely (i.e. 0.05 or 0.01 probability) that such a difference between rates could be observed if the two underlying risks were, in fact, equal. Using the notation P_j _for the *p*-value of the *j*-th test, the decision rule can be expressed as:

Reject H_0 _for unit *v*_*j *_if *P*_*j *_≤ *α*

The significance level α of a test represents the probability of incorrectly rejecting the null hypothesis, that is declaring two risks significantly different when they are, in fact, not different. This wrong decision is known as a "false positive" or a "type I error". In the present application, the test will be repeated for each geographic area under study (e.g. county). As the number of tested areas increases, it becomes increasingly likely that some tests will turn out significant by chance alone (even if the null hypothesis of rate equality is true in all cases). For example, the independent testing of *n *counties under a significance level of α will lead to a (1-(1-α)^n^) probability that at least one test is significant even if none of the *n *counties actually has a rate disparity. This probability is the "experiment-wise" or "family-wise" error rate (FWER) and will increase as the number of tests increases. For *n *= 10 counties and α = 0.05 the probability that a type I error does occur among all 10 hypotheses tested is 0.40, which is much larger than the probability of 0.05 for each test separately that is known as the "comparison-wise" error rate.

Multiple testing corrections reduce the significance level applied to each test so that the overall false positive rate is kept to less than or equal to the user-specified significance level α. Methods to correct for multiple testing differ in their ease of implementation and their stringency. The more stringent or conservative a correction, the fewer false positives are allowed but at the expense of a potential high rate of false negatives (i.e. racial disparities go undetected). The most stringent method is the straightforward Bonferroni correction whereby the significance level is simply divided by the total number *N *of tests, leading to the following decision rule:

Reject H_0 _for unit *v*_*j *_if *P*_*j *_≤ *α*_Bonf _= *α*/*N*

In the context of geographical analyses, where hundreds or thousands of tests can be carried out, this correction quickly becomes excessive and can lead to many missed meaningful findings (false negatives) [17]. For our example with *N *= 688 counties tested, the Bonferroni correction results in a significance level α_Bonf _= 0.000073 = 0.05/688 to detect any significant difference between rates. Sidak [25] proposed a similar correction that proved to be more powerful when the test statistics are independent, which is the case here since each geographic unit is tested independently of the others. The adjusted significance level α_Sidak _is computed as 1-(1-α)^1/N^, leading for the earlier example to a corrected level of 0.000075 = 1-(1-0.05)^1/688^. Sidak's correction thus appears to be as conservative as the Bonferroni correction and so was not investigated further in this paper.

Holm [26] proposed an 'improved Bonferroni procedure' that is less stringent and starts with a ranking of all *p*-values from the smallest P_(1) _to the largest P_(N)_. The magnitude of the α-adjustment then decreases as the rank *k *of the *p*-value increases, i.e. the division factor is (N-k+1). Holm's procedure rejects the null hypothesis of equality of rates for the *j*-th unit if its *p*-value and each of the *p*-values of lower rank are less than the adjusted significance level:

Reject H_0 _for unit *v*_*j *_(P - rank = k) if *P*_(*i*) _≤ *α*/(N - i+1) ∀i = 1,..., k

Similar stepwise procedures were developed afterwards, such as Simes' procedure [27] or the 'extended Simes procedure' [28].

All the methods introduced so far belong to the category of family-wise approaches that tend to produce conservative results. Recently, a set of procedures based on a new criterion called the false discovery rate (FDR) was developed. Instead of controlling the chance of any false positive, one aims to control the expected proportion of true null hypotheses rejected out of the total number of rejections. FDR approaches are thus less restrictive and more powerful than FWER approaches. Benjamini and Hochberg [29] proposed a stepwise FDR procedure for independent tests. Like the 'improved Bonferroni procedure', the first step is to rank all *p*-values by ascending order and apply a correction that decreases as the rank *k *of the *p*-value increases, i.e. the division factor is k/N. The decision rule is however sequential and involves checking that the *p*-value of rank *k *does not exceed the adjusted significance level, starting with the larger *p*-value (k = N). Once this condition has been met for a given rank *k'*, the adjusted significance level α_FDR _is set to k' α/N and applied to all tests of hypothesis. The decision rule can then be formulated as:

Find the largest *k' *= *N*, *N*-1,...,1 such that *P*_(*k*') _≤ *k'α*/*N*

Then Reject H_0 _for all units *v*_j _with P_j _≤ *P*_(*k'*)_

## Results and discussion

### Simulated data sets

For each set of simulated rates, the six types of disparity statistic and the corresponding *p*-values were computed for both one-tailed (Δ_0 _= 0) and two-tailed tests. Differences between WM and BM rates were then declared significant if the *p*-value did not exceed a given significance level α. Two types of errors could occur: misclassification of a county with same underlying WM and BM mortality risk as displaying significant racial disparities (type I error or false positive) and misclassification of a county with actual risk disparities (called target county hereafter) as non-significant (type II error or false negative).

**Figure 3:**
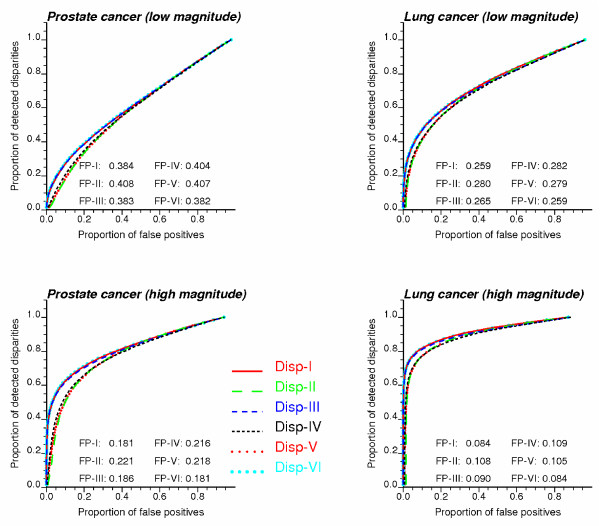
**Receiver Operating Characteristic (ROC) curves for the six disparity statistics**. ROC curves plot the probability of false positive versus the probability of detection. These curves were obtained under two different scenarios for the magnitude of disparities (low = magnitude 1, high = magnitude 4) and a 10% frequency (i.e. 10% of counties have significant disparities). The average proportion of false positives (FP) is listed for each type of disparity statistics (two-tailed tests).

Results were first assessed using the Receiver Operating Characteristic (ROC) curves since they do not require the choice of a particular significance level α and thus are insensitive to the multiple testing corrections. ROC curves plot the probability of false positive versus the probability of detection [30]. The probability of detection corresponds here to the proportion of target counties that are detected with disparities as the significance level increases. In practice, the significance levels are identified with the *p*-values of the target counties. For each of them the probability of false positive is computed as the proportion of non-target counties that are wrongly declared significant. Figure 3 shows the ROC curves obtained for each cancer and six types of disparity statistics (two-tailed tests). Results are plotted for the low and high disparity magnitude classes and the 10% frequency class, and are averaged over all 1,000 realizations (20 simulations of frequency class × 50 simulations of rate values for each scenario).

The most efficient statistic is the one that allows the detection of a larger fraction of target counties at the expense of fewer false positives; that is the ROC curve should be as close as possible to the vertical axis. A quantitative measure of the detection efficiency is the relative area above the ROC curve, which represents the average proportion of false positives (FP). The smaller this value, the better the disparity statistic. As expected, the proportion of false positives tends to increase when the disparities are of small magnitude (top graphs) and the mortality rates are less reliable because of the small number problem (prostate cancer). In all cases, the best results are obtained for the statistics Disp_I _(RD type) and Disp_VI _(RR type), with Disp_III _being a close third for prostate cancer. In other words, the use of the population-weighted average of rates z¯
 MathType@MTEF@5@5@+=feaafiart1ev1aaatCvAUfKttLearuWrP9MDH5MBPbIqV92AaeXatLxBI9gBaebbnrfifHhDYfgasaacH8akY=wiFfYdH8Gipec8Eeeu0xXdbba9frFj0=OqFfea0dXdd9vqai=hGuQ8kuc9pgc9s8qqaq=dirpe0xb9q8qiLsFr0=vr0=vr0dc8meaabaqaciaacaGaaeqabaqabeGadaaakeaacuWG6bGEgaqeaaaa@2E41@*j *instead of individual ethnic rates works best for the rate difference, while the opposite result is found for the relative risk statistic. This ranking is confirmed in Table 2 that lists results obtained for all four classes of disparity magnitude (FP values are averaged over all three frequency classes since it does not affect the results). In addition to the proportions of false positives averaged over all 1,000 simulations, the percentage of simulations where the particular statistic yields the best results is also reported. According to this new criterion, the risk ratio statistic (Disp_VI_) outperforms the other statistics in the most critical scenario, i.e. disparities of smaller magnitude and less reliable rates. The benefit of this statistic is even more pronounced for the one-tailed tests (Δ_0 _= 0), with a percentage of best results of 68–87% for prostate and 63–67% for lung (Table not shown). For one-tailed tests with non-zero thresholds Δ_0_, RD and RR type statistics can not be compared since they correspond to different null hypotheses.

**Table 2:** Average proportion of false positives committed when detecting a significant disparity (two-tailed tests). Calculations are conducted for the six test statistics and four classes of increasing magnitude for the disparity. Numbers into parentheses give the percentage of simulations where the particular statistic yields the smallest proportion of false positives.

	**Disp**_**I**_	**Disp**_**II**_	**Disp**_**III**_	**Disp**_**IV**_	**Disp**_**V**_	**Disp**_**VI**_
**Prostate**						
Magnitude 1	0.384 (21%)	0.408 (1%)	0.385 (32%)	0.405 (1%)	0.407 (0%)	0.382 (45%)
Magnitude 2	0.297 (28%)	0.330 (0%)	0.301 (25%)	0.327 (0%)	0.329 (0%)	0.297 (47%)
Magnitude 3	0.234 (33%)	0.272 (0%)	0.239 (22%)	0.269 (0%)	0.269 (0%)	0.234 (45%)
Magnitude 4	0.186 (40%)	0.225 (0%)	0.191 (19%)	0.222 (0%)	0.222 (0%)	0.186 (41%)
**Lung**						
Magnitude 1	0.256 (48%)	0.277 (0%)	0.263 (6%)	0.279 (0%)	0.276 (0%)	0.257 (46%)
Magnitude 2	0.159 (55%)	0.182 (0%)	0.167 (4%)	0.185 (0%)	0.181 (0%)	0.160 (41%)
Magnitude 3	0.111 (57%)	0.135 (0%)	0.119 (4%)	0.137 (0%)	0.133 (0%)	0.113 (39%)
Magnitude 4	0.081 (56%)	0.105 (0%)	0.088 (4%)	0.106 (0%)	0.102 (0%)	0.083 (40%)

**Figure 4:**
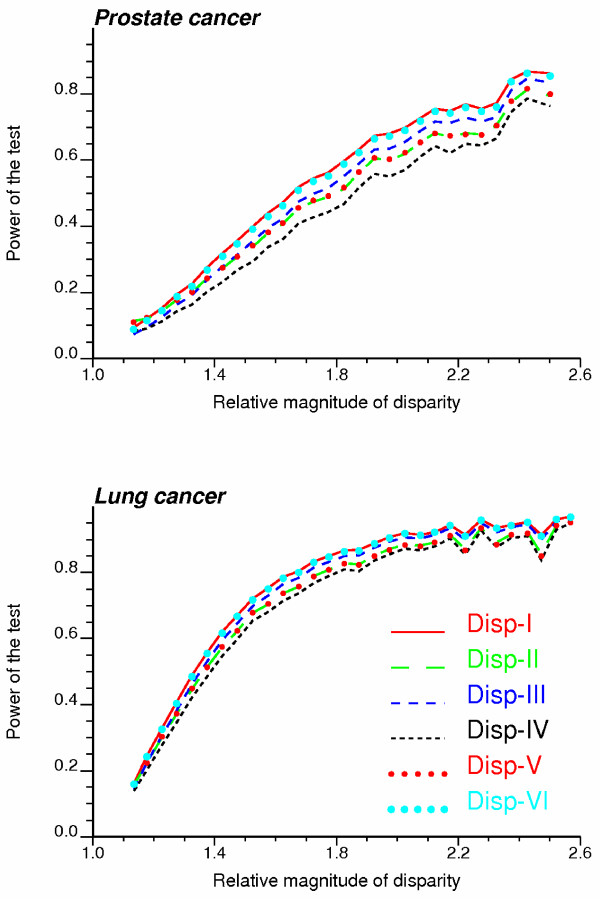
**Power curves for the six disparity statistics**. Each power curve measures the probability of detecting significant disparities as a function of the magnitude of the disparities expressed in terms of risk ratio. The results were obtained over all frequency and magnitude scenarios and using a significance level α = 0.05.

Another important performance criterion is the power of the test, which measures the probability of rejecting the null hypothesis when it is not true (i.e. probability of detecting significant disparities). This probability is expected to be a function of the magnitude of the disparities, and this relationship can be displayed using the so-called power curve. Figure 4 shows the power curves obtained for each cancer and six types of disparity statistics (two-tailed tests with no correction for multiple testing). Results are plotted for a significance level α = 0.05 and the disparity magnitude is expressed in terms of risk ratio. Rates simulated under all scenarios for frequency and magnitude classes are combined. As expected, it is easier to detect disparities of higher magnitude (i.e. higher power); the increase in power is steeper for the more reliable lung cancer mortality rates. For a cancer more likely to be affected by the small number problem, such as prostate cancer mortality, the most powerful tests are systematically the ones based on the statistics Disp_I _(RD type) and Disp_VI _(RR type), which confirms the results obtained for the ROC curves.

Power curves were created for a couple of significance levels and using three methods for multiple testing correction: Bonferroni, Holm, and false discovery rate (FDR). Results were averaged over all magnitude classes and the resulting average power is listed in Table 3. Clearly, the Bonferroni and Holm corrections are too conservative and substantially decrease the power of the test: only a small fraction of target counties are declared significant. The power increases for the less restrictive FDR approach, yet it remains smaller than the power of the uncorrected tests. No matter the type of correction, tests based on the statistics Disp_I _(RD type) and Disp_VI _remain the most powerful. A similar ranking is obtained for the one-tailed tests (Δ_0 _= 0) which tend to be more powerful than the two-tailed tests however. One-tailed tests based on RD or RR-type test statistics cannot be compared for non-zero thresholds Δ_0 _since they correspond to different null hypotheses. Nevertheless, tests based on the statistic Disp_I _are the most powerful among RD types, while Disp_VI _leads to the most powerful test among RR types.

**Table 3:** Average power of two-tailed tests for two significance levels (unadjusted and corrected for multiple testing). Results for the six test statistics and two significance levels are averaged over all classes of disparity magnitude. Significance levels are either unadjusted or reduced using the Bonferroni correction, the Holm's procedure or the False Discovery Rate (FDR) approach.

**Test statistic**	**α = 0.05**				**α = 0.10**			
		
	**Unadj.**	**Bonf.**	**Holm**	**FDR**	**Unadj.**	**Bonf.**	**Holm**	**FDR**
**Prostate**								
Disp_I_	0.371	0.091	0.091	0.175	0.450	0.103	0.103	0.215
Disp_II_	0.328	0.067	0.068	0.153	0.416	0.077	0.077	0.194
Disp_III_	0.333	0.083	0.083	0.154	0.404	0.094	0.094	0.186
Disp_IV_	0.289	0.058	0.059	0.122	0.367	0.067	0.067	0.154
Disp_V_	0.329	0.066	0.067	0.151	0.417	0.076	0.077	0.192
Disp_VI_	0.363	0.083	0.084	0.163	0.443	0.095	0.095	0.201
**Lung**								
Disp_I_	0.386	0.076	0.076	0.157	0.474	0.087	0.088	0.203
Disp_II_	0.353	0.068	0.068	0.143	0.446	0.077	0.077	0.184
Disp_III_	0.362	0.072	0.073	0.144	0.445	0.083	0.083	0.185
Disp_IV_	0.328	0.060	0.060	0.122	0.416	0.068	0.069	0.159
Disp_V_	0.354	0.066	0.067	0.143	0.446	0.076	0.076	0.185
Disp_VI_	0.383	0.074	0.074	0.153	0.471	0.085	0.085	0.198

For all methods, the power of the test increases for larger significance levels α since it becomes easier to reject the null hypothesis; see Table 3. The trade-off cost is the increase in false positives which is acceptable as long as that proportion of false positives is close to the significance level α. The proportion of false positives was computed for the same scenarios considered for the power evaluation in Table 3. Table 4 indicates that for both α levels the best agreement is found for uncorrected tests using the statistic Disp_I _(RD type), with the risk ratio statistic Disp_VI _being a close second. Multiple testing correction strongly reduces the risk of false positives, yet at the expense of a lower power as demonstrated in Table 3.

**Table 4:** Average proportion of false positives for two-tailed tests (unadjusted and corrected for multiple testing). Results for the six test statistics and two significance levels are averaged over all classes of disparity magnitude. Significance levels are either unadjusted or reduced using the Bonferroni correction, the Holm's procedure or the False Discovery Rate (FDR) approach.

**Test statistic**	**α = 0.05**				**α = 0.10**			
		
	**Unadj.**	**Bonf.**	**Holm**	**FDR**	**Unadj.**	**Bonf.**	**Holm**	**FDR**
**Prostate**								
Disp_I_	0.048	0.0	0.0	0.002	0.096	0.001	0.001	0.005
Disp_II_	0.093	0.025	0.025	0.039	0.139	0.027	0.027	0.047
Disp_III_	0.032	0.0	0.0	0.001	0.065	0.0	0.0	0.002
Disp_IV_	0.063	0.012	0.012	0.020	0.098	0.013	0.013	0.024
Disp_V_	0.091	0.016	0.016	0.031	0.139	0.018	0.018	0.039
Disp_VI_	0.042	0.0	0.0	0.001	0.087	0.0	0.0	0.003
**Lung**								
Disp_I_	0.049	0.0	0.0	0.004	0.098	0.0	0.0	0.009
Disp_II_	0.068	0.016	0.016	0.022	0.115	0.016	0.016	0.028
Disp_III_	0.039	0.0	0.0	0.003	0.079	0.0	0.0	0.006
Disp_IV_	0.054	0.009	0.009	0.014	0.095	0.009	0.009	0.018
Disp_V_	0.068	0.012	0.012	0.021	0.116	0.013	0.013	0.027
Disp_VI_	0.047	0.0	0.0	0.003	0.095	0.0	0.0	0.008

### Cancer data sets

County-level disparities in the cancer mortality maps of Figure 1 were investigated using the different types of disparity statistics and multiple testing corrections. Table 5 reports the number of counties where disparities tested significant for the statistics Disp_I _(RD type) and Disp_VI _(RR type) which performed best in the simulation studies. These results illustrate the much wider extent of racial disparities for prostate cancer versus lung cancer: 4 to 15 times more counties tested significant for prostate cancer. For all scenarios, the risk ratio statistic (Disp_VI_) leads to slightly fewer significant counties which form a subset of the counties flagged using the risk difference statistic (Disp_I_). As expected, multiple testing correction reduces the proportion of significant tests. This reduction is particularly large when disparities are of smaller magnitude (i.e. lung cancer) and so the *p*-values of the tests are larger. Conversely, the unadjusted test and the False Discovery Rate approach lead to very similar results for prostate cancer where the average mortality rate for black males is twice the rate for white males.

**Table 5:** Number of counties where the difference between cancer mortality rates is declared significant (two-tailed test). Results are reported for the two test statistics that performed best in simulation studies. Significance levels are either unadjusted or reduced using the Bonferroni correction, the Holm's procedure or the False Discovery Rate (FDR) approach. Agreement refers to the number of counties declared significant by both tests.

**Test statistic**	**α = 0.01**				**α = 0.05**			
		
	**Unadj.**	**Bonf.**	**Holm**	**FDR**	**Unadj.**	**Bonf.**	**Holm**	**FDR**
**Prostate**								
Disp_I_	385	176	184	362	462	213	222	430
Disp_VI_	372	167	170	350	452	200	209	422
Agreement	372	167	170	350	452	200	209	422
**Lung**								
Disp_I_	70	13	13	26	136	19	19	48
Disp_VI_	66	13	13	25	134	19	19	48
Agreement	66	13	13	25	134	19	19	48

**Figure 5:**
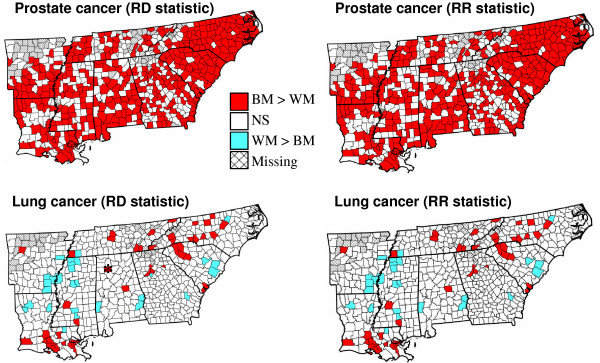
**Maps of counties with significant racial disparities for prostate and lung cancer mortality rates**. Two-tailed tests based on the statistics Disp_I _(RD type) and Disp_VI _(RR type) were conducted at a significance level α = 0.01. White polygons depict non-significant (NS) differences, while hatched areas correspond to missing data. Thicker lines delineate state boundaries. Significant disparities are color coded according to the sign of the difference. The star in the lung cancer map indicates Winston County where the RD and RR statistics lead to different conclusions.

Counties detected at the significance level α = 0.01 (unadjusted tests) are displayed in two colors in the maps of Figure 5. Red is used to depict counties where BM mortality rates exceed WM rates, information that is lost in two-tailed tests. For lung cancer, one of the four counties that test significant for the risk difference but not the risk ratio is Winston County in Alabama; see Figure 5 (star in left bottom map). The mortality rate is 69.9 per 100,000 person-years for white males and 376.7 per 100,000 person-years for black males. The very high BM rate is likely unreliable given the small size of the population at risk: 616 versus 268,837 for white males. In this case, the risk ratio statistic (Disp_VI_) assigns more importance to the lack of reliability of the extreme rate than the extent of the difference between rates.

**Figure 6:**
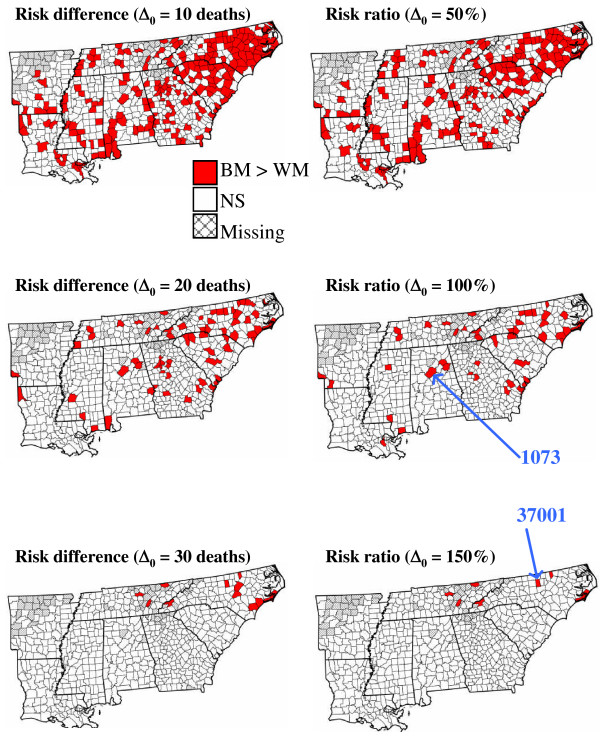
**Maps of counties with significantly greater prostate cancer mortality rates for black males**. The threshold difference Δ_0 _is expressed either in absolute term (deaths/100,000 habitants) or percentage, depending on the type of statistic used (RD versus RR type). One-tailed tests were conducted at a significance level α = 0.01. White polygons depict non-significant (NS) differences, while hatched areas correspond to missing data. Thicker lines delineate state boundaries. The pointing arrows in the right maps indicate the two counties (Alamance, NC: FIPS 37001, Jefferson, AL: FIPS 1073) with the smallest *p*-value for the one-tailed tests.

Even at the very low significance level α = 0.01, more than half the counties tested significant for disparities in prostate cancer mortality. The difference between rates is thus so large and systematic that two-tailed tests are not highly informative. One-tailed tests were used to identify subsets of counties where the BM mortality rates exceed WM rates by an amount that is significantly greater than a given threshold Δ_0_. Three absolute thresholds were used for one-tailed tests based on the risk difference statistic (Disp_I_): 10, 20 and 30 deaths/100,000 habitants. For the risk ratio statistic Disp_VI_, relative thresholds of similar magnitude (recall that the population weighted mean for WM rates is 21.7 per 100,000 person-years) were chosen as: 0.5, 1 and 1.5. In other words, one tested the following null hypotheses: the BM rate is 50%, 100% or 150% greater than the WM rate recorded in the same county. Results displayed in Figure 6 allow a finer analysis of the spatial distribution of racial disparities. In particular they reveal a few counties in North Carolina and Tennessee where the disparities are the largest. For both the 30 death and 150% thresholds, the largest test statistic was found for Alamance County (North Carolina): the WM mortality rate is 21.35 per 100,000 person-years and the BM rate is 72.77 per 100,000 person-years. Although other counties display greater disparities in absolute or relative terms, Alamance County has a larger population at risk, which makes the rates more reliable and hence smaller differences are easier to detect. For the other thresholds, the largest test statistic was found for the most densely populated county in Alabama: Jefferson, the county seat being Birmingham. Once again the racial disparity is not the most extreme (WM rate: 22.32, BM rate: 53.90) but these rates are derived from large population sizes, which greatly reduces their uncertainty.

## Conclusion

Too often racial disparities are evaluated simply by computing the difference between crude rates, ignoring the lack of reliability of rates recorded for small minority populations. The small number problem is even more pronounced for diseases with low frequency of occurrence (e.g. mortality rates for rare cancers or cancers with high survival rate). Temporal change in rate differences is also an incomplete measure of the progress towards the elimination of disparities [3]. The assessment of significant racial disparities across geographic areas is an important tool in guiding cancer control practices, and public health officials must consider the problems of small population size and multiple comparison, and should conduct disparity analyses using both absolute (difference, RD statistic) and relative (ratio, RR statistic) measures.

The test statistics introduced in this paper incorporate the population size directly into the relative or absolute comparison of rates. These are well-known [23,24] test statistics which have mainly been used in a non-spatial context thus far. Their application to a set of geographic areas requires one to consider the potential impact of multiple testing on the rate of false positives. This paper reviewed traditional (i.e. Bonferroni or Holm) and more recent approaches (False Discovery Rate) for multiple testing correction.

According to our simulation studies, two statistics (RD and RR type) systematically provided the largest power across all scenarios for the frequency and magnitude of disparities. Analysis of ROC curves showed the same two statistics generated the smaller average proportion of false positives. Best results (i.e. higher power and fewer false positives) were obtained for simulations based on lung cancer mortality rates since they are less affected by the small number problem than prostate cancer. Somewhat surprisingly, multiple testing did not appear to be an issue and the unadjusted tests yielded the expected proportion of false positives. Fewer false positives were observed after multiple testing correction, yet the trade-off cost is the loss of power which is particularly strong for conservative methods, such as Bonferroni and Holm corrections. The FDR approach yields intermediate results in terms of power and false positives and could be a valuable alternative to unadjusted tests if false positives are deemed much costlier than false negatives. If a predicted level of random false positives is preferred, unadjusted tests are recommended for the disparity statistics introduced in this paper.

These recommendations are based on a necessarily restricted set of simulation scenarios and should be refined in future studies based on other distributions for cancer rates and smaller geographies (e.g. ZIP codes) where the small number problem is more pronounced. The present research considered only the simple case of detecting disparities among two ethnic groups (i.e. black and white males). Given the increasing racial/ethnic diversity of the US and the availability of race-specific health outcomes, there is the need for methods to detect disparities across three or more population groups. Keppel et al. (2004) introduced an index of disparity [2] that summarizes differences between the rates of several groups and a "reference" rate which can be the rate for the group with the most favorable health outcome, the total population rate, or a predefined target. Standard errors for the index of disparity are obtained using a type of resampling or "bootstrap" procedure. This index was used to detect disparities in infant mortality rates among six race and ethnic groups. To identify which specific groups differ significantly, this global measure of disparity should be supplemented with the pairwise comparisons of rates introduced in this paper. Research is needed to investigate the impact of the repetition of tests both in space and across population groups on the multiple comparison problem.

The analysis of lung and prostate cancer mortality maps illustrates the usefulness of test statistics to quickly identify counties with significant disparities and how one-tailed tests allow one to consider more specific null hypotheses. The incorporation of such tests in user-friendly software should improve our ability to interpret geographic variation in cancer disparities, detect changes in space (e.g. cluster of counties with significant disparities) and through time (e.g. change in health disparities following strategies to improve cancer prevention and early detection), and to better understand the causes underlying observed racial disparities in cancer incidence, mortality and morbidity.

In the future, the straightforward statistics presented in this paper will be compared to formulations where mortality risks and the associated standard errors are estimated using a model-based approach (e.g. Poisson or Bayesian methods) that capitalizes on the spatial correlation between rates measured in neighboring units. In particular, this interpolation-based approach should allow the detection and mapping of health disparities for small geographies with a high frequency of missing data caused by the small number of cases reported.

## Competing interests

The authors are affiliated with BioMedware a research company that also develops software for the exploratory spatial and temporal analysis of health and environmental data. With funding from the National Cancer Institute, the authors developed STIS (Space-Time Intelligence System), which is a commercial product of Terraseer that will include these disparity statistics and multiple testing corrections in a future release.

## Authors' contributions

PG carried out all simulation studies and drafted the manuscript. JRM contributed to the literature review of the disparity statistics. JRM and GMJ participated in the design of the study. All authors read and approved the final manuscript.
